# Estimating copy numbers of alleles from population-scale high-throughput sequencing data

**DOI:** 10.1186/1471-2105-16-S1-S4

**Published:** 2015-01-21

**Authors:** Takahiro Mimori, Naoki Nariai, Kaname Kojima, Yukuto Sato, Yosuke Kawai, Yumi Yamaguchi-Kabata, Masao Nagasaki

**Affiliations:** 1 Department of Integrative Genomics, Tohoku Medical Megabank Organization, Tohoku University, 2-1 Seiryo-machi, Aoba-ku, Sendai, Miyagi, Japan; 2 Department of Integrative Genomics, Tohoku Medical Megabank Organization, Tohoku University, 2-1 Seiryo-machi, Aoba-ku, Sendai, Miyagi, Japan

**Keywords:** copy number variation, high-throughput sequencing data, latent Dirichlet allocation

## Abstract

**Background:**

With the recent development of microarray and high-throughput sequencing (HTS) technologies, a number of studies have revealed catalogs of copy number variants (CNVs) and their association with phenotypes and complex traits. In parallel, a number of approaches to predict CNV regions and genotypes are proposed for both microarray and HTS data. However, only a few approaches focus on haplotyping of CNV loci.

**Results:**

We propose a novel approach to infer copy unit alleles and their numbers in each sample simultaneously from population-scale HTS data by variational Bayesian inference on a generative probabilistic model inspired by latent Dirichlet allocation, which is a well studied model for document classification problems. In simulation studies, we evaluated concordance between inferred and true copy unit alleles for lower-, middle-, and higher-copy number dataset, in which precision and recall were ≥ 0.9 for data with mean coverage ≥ 10× per copy unit. We also applied the approach to HTS data of 1123 samples at highly variable salivary amylase gene locus and a pseudogene locus, and confirmed consistency of the estimated alleles within samples belonging to a trio of CEPH/Utah pedigree 1463 with 11 offspring.

**Conclusions:**

Our proposed approach enables detailed analysis of copy number variations, such as association study between copy unit alleles and phenotypes or biological features including human diseases.

## Background

With the recent development of microarray and high-throughput sequencing (HTS) technologies, extensive efforts have elucidated catalogs of haplotypes and genomic variations such as single nucleotide polymorphisms (SNPs), indels, copy number variations (CNVs) and other structural variations in population [1-3]. Based on these catalogs of genomic variations and haplotype structures, a number of genome wide association studies (GWAS) have been conducted to identify associations between genomic variations and phenotypes.

Recent studies also revealed that CNVs affect phenotypes and complex traits, such as human diseases [4-8]. In parallel, a number of methods for detecting CNV loci and inferring copy numbers at each CNV locus have been proposed for both microarray and HTS technologies [9-13]. In particular, high coverage and PCR-free sequencing data enable us to estimate copy numbers of CNVs at higher resolution than former technologies because of its quantitative stability. Even for deletions, which are losses of genomic regions with various size, it requires sequencing data with 20× to 30× depths per diploid genomes for accurate detection [13,14].

Not only an absolute copy number, but also characteristics of each copy unit at CNV locus are expected to provide critical information about genetic structure and biological function of the locus. For example, nonsynonymous mutations on coding regions are known to affect biological functions. Hence, identifying these copy units is essential for understanding biological effects of CNVs.

**Figure 1:**
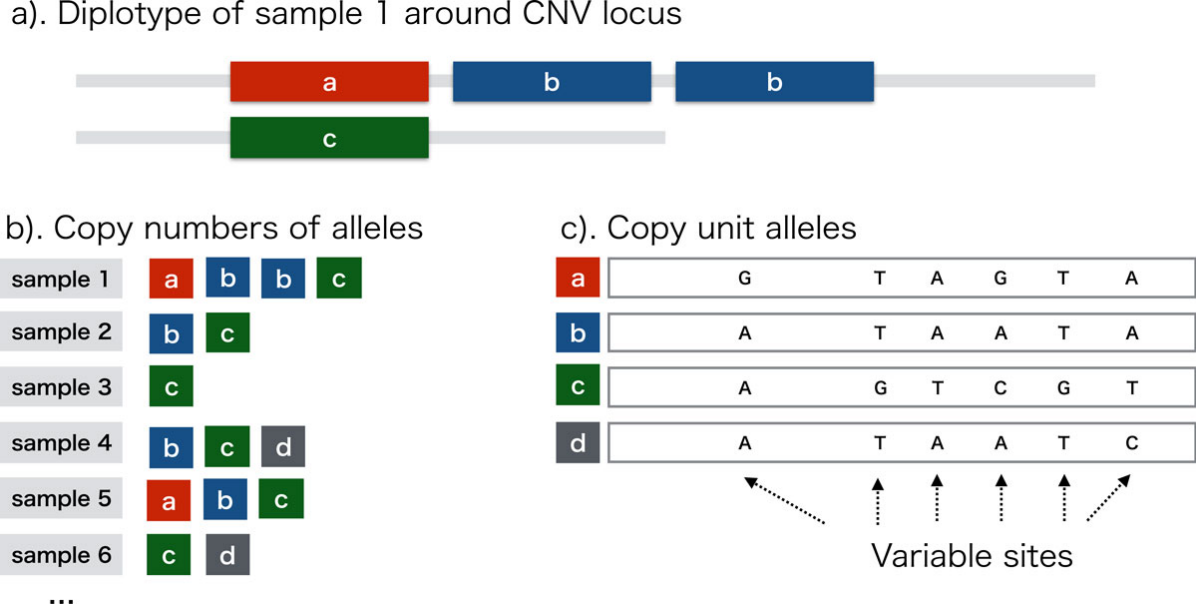
**Illustration of the copy unit alleles and their distributions in a population**. **a**). A CNV locus of diploid genome of sample 1 who has one copy of allele *a*, two copies of allele *b*, and one copy of allele *c *at the locus, **b**). Alleles of each sample are shown, **c**). Four copy unit alleles at a CNV locus are aligned. Difference among these alleles is characterized by bases at variable sites.

The difference among copy units is characterized by haplotypes of *variable sites *in the units (Figure 1) that are supposed to be introduced by mutations during evolutional history in population. If those sequences are similar to each other, *i.e.*, a ratio of mutated bases among all the bases are less than ten percent, then alignment of reads to the reference genome using tools such as BWA [15] will yield an information including the number of mismatched bases observed at variable sites of the CNV locus. This information reflects copy numbers of each copy unit in sequenced samples.

There are some difficulties in determining sequence and copy numbers of each copy unit at CNV locus from sequenced data of samples. First, observable counts of bases come from diploid sequences for autosomal chromosomes, and the true combination of bases at multiple heterozygotes sites is not apparent from the data. Second, at CNV loci, because copy unit alleles are similar to each other, reads are aligned to the same locus of the reference genome. These problems complicate the task to infer sequences and copy numbers of copy units for each sample from read alignment data. The former problem is called phasing, and several approaches to infer haplotypes from SNP and indel genotypes of multiple samples are developed [16-18]. Recently, phasing approaches of an another type which utilize co-occurrence of multiple heterozygote variants on HTS read are devised [19,20]. In particular, HapMonster [20] performs simultaneous estimation of haplotype phasing and variant calling and succeeds in improving both of their performances, which suggests that treating genotype and haplotype with a unified statistical model is promising approach. In contrast to a number of phasing approaches have been devised today, there are only a few approaches for inferring haplotypes of the variable sites in CNV locus [21-23] and they all use microarray data of population as input data. There seems to be no approaches for this task from sequencing data at present, which might be due to a lack of PCR-free, high quality, and high coverage sequencing data of population.

In this study, we propose a novel approach to estimate sequence and copy numbers of copy unit at CNV locus from population-scale sequencing data. In the proposed approach, we construct a generative model of sequenced reads and estimate copy unit sequences and their copy numbers for each sample simultaneously using the expectation maximization (EM) algorithm and the variational Bayesian (VB) inference. The similar models and techniques have been studied in topic models of natural language processing [24,25]. Recently, several bioinformatics approaches such as TIGAR [26] applies the VB inference to estimate transcript isoform abundance from RNA-Seq data.

Due to a limited resolution in identifying allelic ratio with microarray data, previous approaches have been applied to CNVs whose copy numbers are less than or equal to four per diploid [21-23]. On the contrary, our probabilistic model can analyze higher copy number loci with high coverage HTS data, in which the computational complexity is linear with respect to the number of samples and the number of copy unit alleles.

We verified performance of the approaches in simulation studies with various configurations of copy numbers. In a real data analysis, we apply our methods to HTS data of 1123 samples at highly variable salivary amylase gene locus and a pseudogene locus. We also confirmed consistency in predicting copy numbers of copy unit alleles for CEPH trio samples with 11 offspring.

## Methods

### Preprocessing

A precondition is that there is sequenced read data of *N *individual samples, and the read data is aligned to reference sequences that represent sequences at predefined CNV loci. We assume that each sample has combinations of *K *copy unit alleles with which each one has copy number more than or equal to zero at the CNV loci. From aligned data of multiple samples, we can identify *M *variable sites of the alleles to distinguish them.

### The generative model

We construct a generative model of aligned reads at variable sites of CNV loci. In the model, each observed base of the *n*-th sample is assumed to be generated from one of *K *alleles that follows *K *dimensional multinomial distribution with the parameters *θ_n_*, and the base observation probability at the variable site *x *from allele *k *follows multinomial distribution with the parameters *ϕ_kx_*. We also introduce Dirichlet priors ***α ***for parameters *θ_n_*.

The joint probability of observed bases ***b ***and the hidden variables ***z ***and ***θ ***given parameters ***α ***and ***ϕ ***is decomposed as follows:

$$\begin{array}{ccc} P\left(b,z,\theta |\alpha ,\varphi \right) & = & \overset{N}{\underset{n=1}{\prod }}P\left(b_{n}|z_{n},\varphi \right)P\left(z_{n}|\theta_{n}\right)P\left(\theta_{n}|\alpha \right)\\ & = & \overset{N}{\underset{n=1}{\prod }}\left(\overset{M}{\underset{x=1}{\prod }}\overset{d_{nx}}{\underset{t=1}{\prod }}P\left(b_{nxt}|z_{nxt},\varphi \right)P\left(z_{nxt}|\theta_{n}\right)\right)P\left(\theta_{n}|\alpha \right), \end{array}$$

where *b_nxt _*denotes the *t*-th observed base at variable site *x *of sample *n*, which is one of the nucleotide characters: Λ = {*A, T, C*, *G*}, *z_nxt _*denotes the allele index which generates the base *b_nxt_*, and *d_nx _*denotes the number of observed bases at site *x *of sample *n*. The three terms in this equation are calculated as follows:

$$\begin{array}{c} P\left(b_{nxt}=b|z_{nxt}=k,\varphi \right)=\varphi_{kxb},\\ P\left(z_{nxt}=k|\theta_{n}\right),=\theta_{nk},\\ P\left(\theta_{n}|\alpha \right)\equiv \text{Dir}\left(\theta_{n}|\alpha \right)=\frac{\Gamma \left(\underset{k}{\sum }\alpha_{k}\right)}{\underset{k}{\prod }\Gamma \left(\alpha_{k}\right)}\overset{K}{\underset{k=1}{\prod }}\theta_{nk}^{\alpha_{k}-1}, \end{array}$$

where Γ is the gamma function. In this study, we use *α_k _*= 1 (*k = *1*... K*), which assumes uniform priors for *θ_n_*.

### The EM algorithm and the VB inference

We estimate the posterior distribution of the hidden variables *z *and *θ *and a parameter vector ***φ ***which describes emission probability of each base for given variable site *x *and allele *k*, and calculate the marginal log likelihood:

L(X|Φ)≡logP(X|Φ)= ∫ logP(X,Y|Φ)P(Y|Φ)dY,

where *X = *{*b*} is data, *Y = *{*z, θ*} denotes hidden variables, and ***Φ ***= {***φ***} denotes parameters to be estimated.

According to the EM algorithm framework, we can maximize lower bound of the log likelihood by estimating the posterior *P*(***Y***\***X***, ***Φ***) for given parameters ***Φ ***(E step) and maximizing the lower bound by varying parameters ***Φ ***for given posterior distribution (M step) iteratively. In the latter M step, we further approximate the posterior distribution with factorized functions:

$$\begin{array}{ccc} Q\left(Y\right) & = & Q\left(z,\theta \right)=Q\left(z_{1}...z_{N},\theta_{1}...\theta_{K}\right)=\left(\overset{N}{\underset{n=1}{\prod }}\overset{M}{\underset{x=1}{\prod }}\overset{d_{nx}}{\underset{t=1}{\prod }}Q\left(z_{nxt}\right)\right)\left(\overset{K}{\underset{k=1}{\prod }}Q\left(\theta_{k}\right)\right),\\ Q\left(z_{nxt}\right) & \equiv & \text{Multinom}\;\left(z_{nxt}|w_{n}\right)=\underset{k}{\prod }w_{nk}^{I\left(z_{nxt}=k\right)},\\ Q\left(\theta_{n}\right) & \equiv & \text{Dir}\left(\theta_{n}|r_{n}\right)=\frac{\Gamma \left(\underset{k}{\sum }r_{nk}\right)}{\underset{k}{\prod }\Gamma \left(r_{nk}\right)}\underset{k}{\prod }\theta_{nk}^{r_{nk}-1}, \end{array}$$

where we introduce new parameters ***w ***and ***r ***to describe the approximate distributions of *z *and *θ*, respectively, and *I *(statement) denotes the indicator function which equals to 1 if the statement is true, otherwise 0.

In the E step, we update the parametrized function *Q *iteratively according to following formulas to maximize the lower bound *L:*

(1) $$w_{nxtk}\propto \varphi_{kx}b_{nxt}\text{exp}\left[\Psi \left(r_{nk}\right)-\Psi \left(\underset{k}{\sum }r_{nk}\right)\right],$$

(2) $$rnk=\overset{M}{\underset{x=1}{\sum }}\overset{d_{nx}}{\underset{t=1}{\sum }}w_{nxtk}+\alpha_{k},$$

where Ψ is the digamma function, which is the first derivative of the log Gamma function.

In the M step, we update the parameters ***φ ***according to following formula:

(3) $$\varphi_{kxb}\propto \overset{N}{\underset{n=1}{\sum }}\overset{d_{nx}}{\underset{t=1}{\sum }}I\left(b_{nxt}=b\right)w_{nxtk}.$$

### Computational complexity

In the E step, Eq. (1) shows that *w_nxtk _*depends on *t *only through the observed base *b_nxt_*. From this fact and Eq. (2) and Eq. (3), it is clear that the computational complexity of each iteration is *O*(*NMK*|Λ|), where |Λ| is the number of possible bases. One of the ways to determine the number of alleles *K *is that, estimate the marginal log likelihood of the model for each value *K *with its lower bound and select the most likely value $\;\hat{K}$ which provides highest one.

## Results and discussion

### Simulation analysis 1

#### Data preparation

In the simulation analysis 1, we set the number of copy unit alleles four, the number of variable sites at CNV region *M = *16, nucleotide bases of these sites as shown in Table 1 the number of samples *N = *12 with which four samples have two alleles, another four have three alleles, and the remainder have four alleles, respectively. Each allele of samples is chosen with equally probability from the four alleles. From these samples, we generate histogram of bases at the variable sites that correspond to aligned HTS read data. The number of observed read at each variable site follows a mixture of Poisson distribution for each copy unit allele and their means are set to 15 in this analysis. We also take into account 1% sequencing errors that mutates the correct base to one of the other three bases.

**Table 1:** Copy unit alleles and their bases at variable sites used in simulation analysis 1.

**No**.	Bases at variable sites
1	ATTGCGATATTGCGAT
1	ACGGATTTACGGATTT
3	CTTCGGAACTTCGGAA
4	CGATTGAACGTCGTAC

For simplicity, we assume that all the bases at variable sites have bases taken from one of four characters: A, T, C, and G.

#### Evaluation of the results

**Figure 2:**
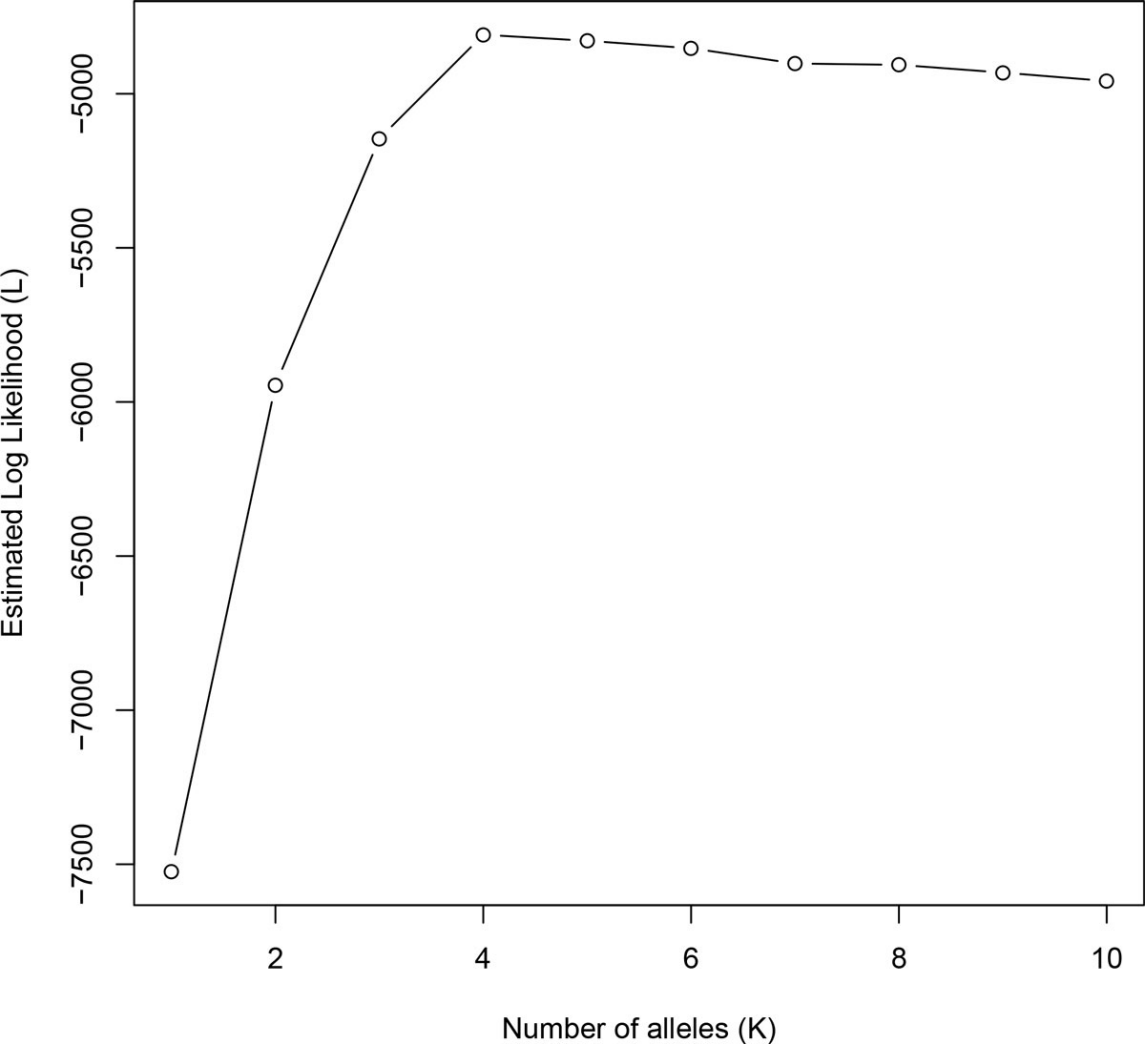
**Lower bound of log likelihood in simulation analysis 1**. Estimation of log likelihood by its lower bound in simulation analysis 1 against variable number of alleles *K*. The true number of alleles *K *= 4 is correctly predicted by maximizing the log likelihood.

We estimated that the number of alleles *K *as four, which maximized log likelihood *L *as shown in Figure 2. For evaluation of allele concordance between true and predicted set, we defined precision and recall of the predictions as follows:

(4) $$\text{precision}\equiv \overset{K}{\underset{k=1}{\sum }}\frac{{\text{max}}_{l\in \left\{1...K_{0}\right\}r_{kl}}}{K},$$

(5) $$\text{recall}\equiv \overset{K_{0}}{\underset{k=1}{\sum }}\frac{{\text{max}}_{l\in \left\{1...K\right\}r_{lk}}}{K_{0}},$$

where *K*_0 _is the number of true alleles which equals to four in this case and *r_kl _*represents the ratio of matched bases at variable sites between the predicted allele *k *and the true allele *l*. The concrete definition of *r_kl _*is as follows:

$$r_{kl}=\frac{\sum_{x=1}^{M}I\left({\hat{b}}_{kx}=b_{lx}^{\left(0\right)}\right)}{M},$$

where ${\hat{b}}_{kx}\equiv \underset{b\in \Lambda }{\arg\max}\varphi_{kxb}$ is a predicted base at variable site *x *of the predicted allele *k *and $b_{lx}^{\left(0\right)}$ is a base at variable site *x *of the true allele *l*.

**Figure 3:**
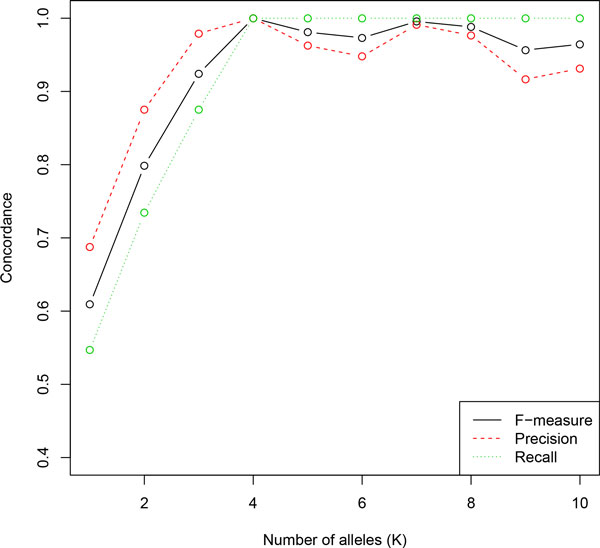
**Allele concordance in simulation analysis 1**. The precision and recall of inferred allele bases at variable sites are both maximized at true number of alleles *K *= 4.

We verified that at *K = K*_0_, precision and recall are both maximized as shown in Figure 3.

### Simulation analysis 2

#### Data, preparation

In this analysis, we used phased haplotypes of 45 males in CEU population released in November 23, 2010 by the 1000 Genomes project [3]. We extract haplotype sequences in a region of 10, 000 bp length at chrX:2, 800,001-2, 810,000 of the hg19 reference genome. The region contains nine distinct haplotypes and 21 variable sites in the population. We generate three different datasets from these haplotypes, that simulate a) lower-, b) middle-, and c) higher-copy number alleles. Copy numbers of alleles in each dataset are summarized in Table 2 which are determined so that the total number of copy units in sample alleles equals to 45. Copy unit alleles in these datasets are randomly chosen from the 45 haplotypes of the region without replacement. We generate histogram of bases at the variable sites as the same way as in the simulation analysis 1, except for various mean depth of coverage that is 3×, 5×, 10×, 15×, and 20× for each copy unit allele from these datasets.

**Table 2:** Configurations of copy numbers and number of samples in three datasets used in simulation analysis 2.

Dataset	List of copy numbers and number of samples
a) lower-copy number	1:5, 2:11, 3:6
b) middle-copy number	2:2, 3:3, 4:4, 5:2, 6:1
c) higher-copy number	3:1, 4:2, 5:3, 6:2, 7:1

For instance, a) lower-copy number set contains five samples with one copy, 11 samples with two copies, and six samples with three copies, respectively. Configurations of datasets b) middle-copy number and c) higher-copy number are shown in the same way.

#### Evaluation of the results

**Figure 4:**
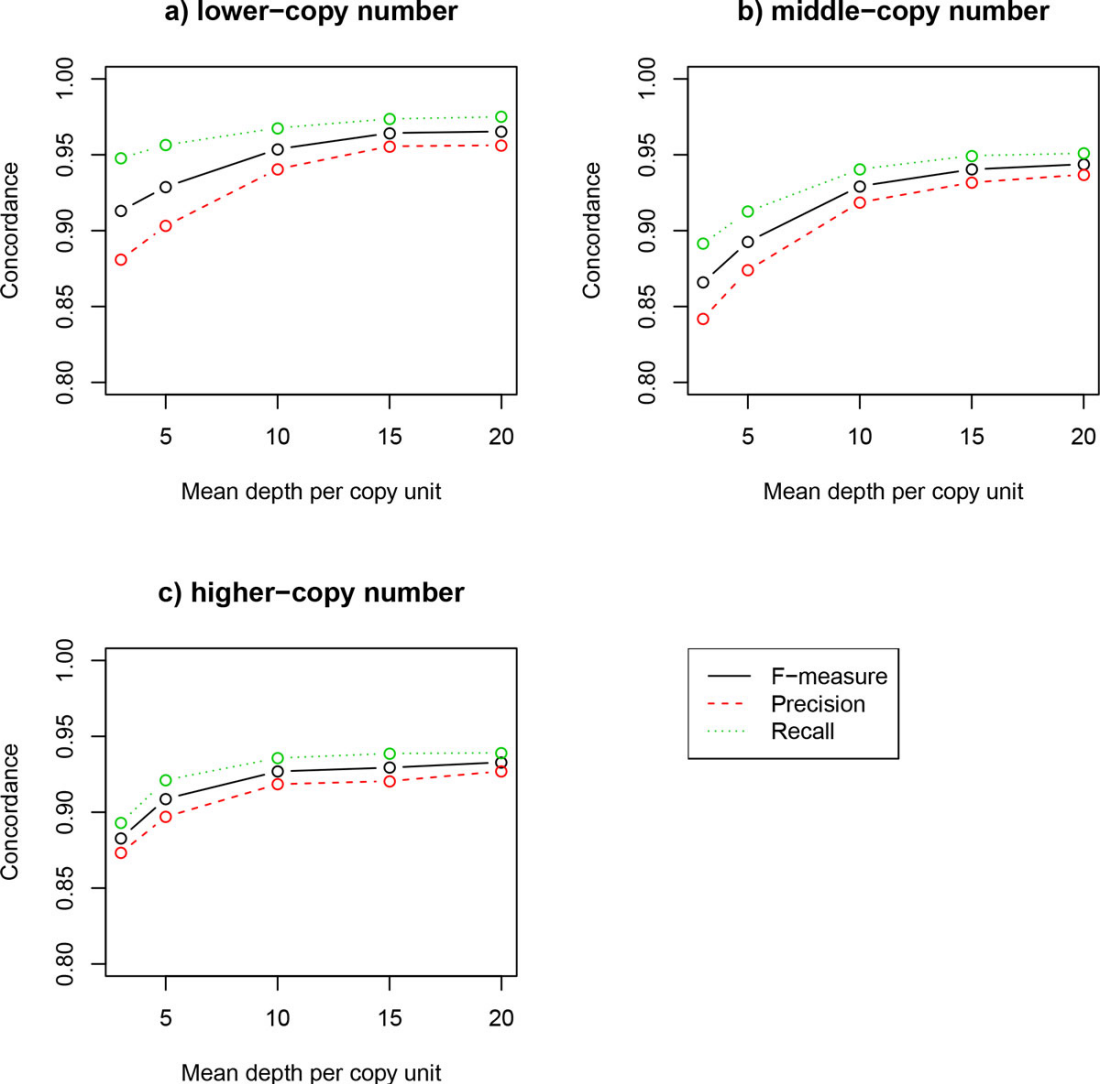
**Allele concordance in simulation analysis 2**. The precision, recall, and F-measure of inferred allele bases at variable sites are shown for three datasets that simulate a) lower-, b) middle-, and c) higher-copy number alleles. As expected, the performance is consistently increased along to mean depth per copy unit in all datasets.

We compare allele concordance for three datasets and varying mean depth of coverage in terms of precision and recall that are defined in Eq. (4) and Eq. (5) respectively. For each dataset and mean depth of coverage, we apply the proposed approach to 100 independently generated histogram of bases at variable sites. Then, we take means of precision, recall, and F-measure which is a harmonic mean of precision and recall, for these replicated data. From the results in Figure 4, we denote that allele concordance is consistently improved by increasing mean coverage of depth. It is also noted that, although a dataset with higher copy numbers is more difficult for accurate estimation than with lower copy numbers as expected, our approach achieves allele concordance > 0.9 in terms of precision, recall, and F-measure with sufficient mean depth of coverage, such as 10x per copy unit.

### Real data application

#### Data, preparation

We estimate copy numbers of copy unit alleles at salivary amylase gene (AMY1) locus using publicly available HTS data of 1123 samples, in which 17 are high coverage data around 50× per diploid genome of Coriell CEPH/Utah pedigree 1463 provided by Illumina's Platinum Genomes project [27] and 1106 are low coverage data around 4× per diploid genome released from the 1000 Genomes project [3]. AMY1 is known as a CNV locus with highly variable copy numbers [28], whose typical copy number is six to ten.

We obtained BAM files, in which HTS reads were aligned to the hgl9 reference sequence. We extracted paired-end reads in FASTQ format that aligned to amylase gene locus chrl:104,129, 283-104, 320, 531. Then, we aligned the extracted reads with BWA [15] to a custom reference sequence that is comprised of extracted sequences of gene coding loci of AMY1A and AMY2A from the hgl9 reference sequence. After the alignment process, we identify 57 variable sites within 835-th to 9200-th bases of AMY1A locus in the 17 high coverage samples. To determine these variable sites, we adopted criterion that observed counts of minor bases ≥ 15 at least one of the 17 samples. For simplicity of analysis, we omitted variable sites that contained deletions whose observed ratio is ≥ 0.1 against the total observed bases at the same sites.

#### Evaluation of the results

**Figure 5:**
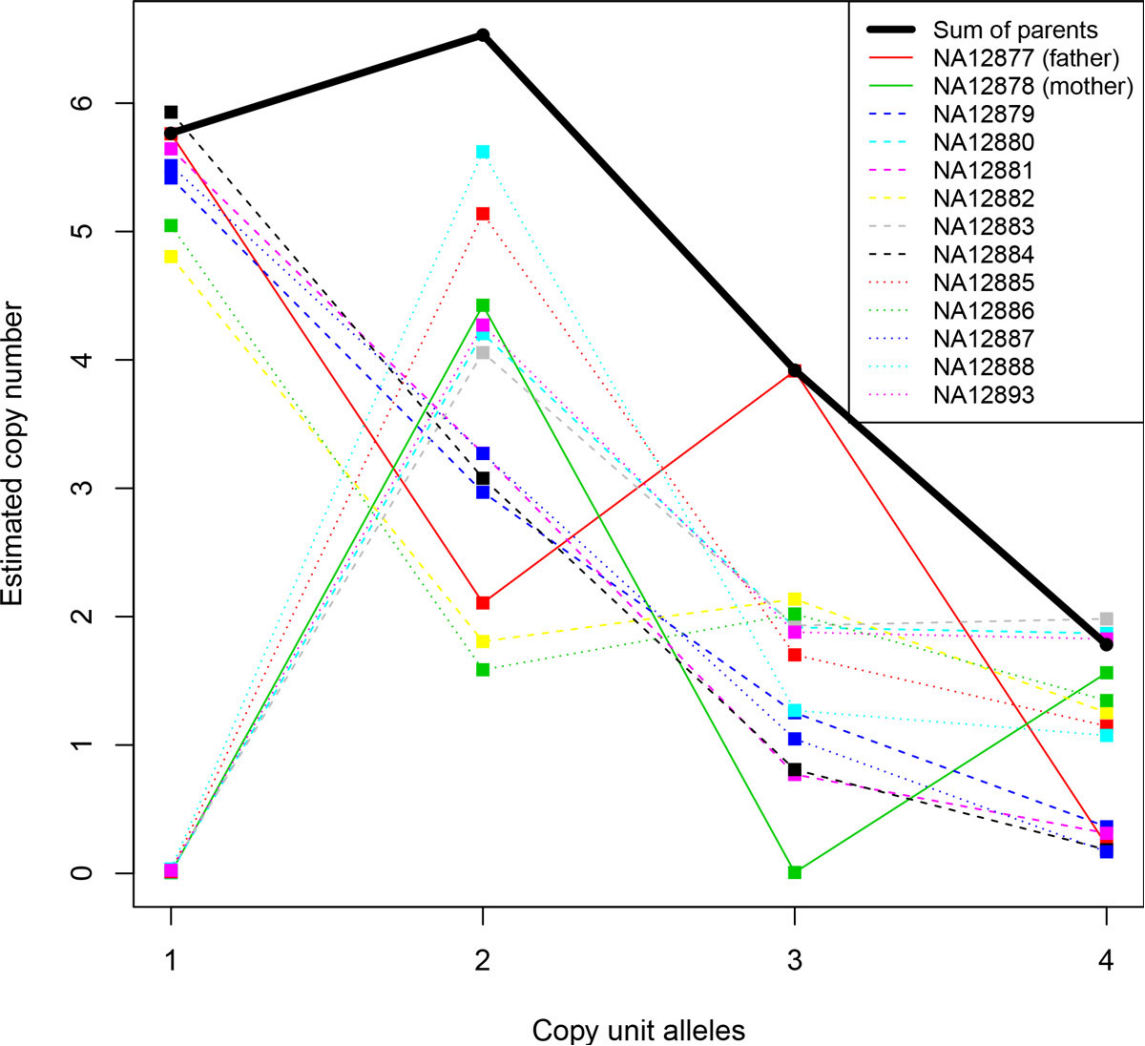
**Estimated copy number of each allele at AMY1 gene locus in real data analysis**. Estimated copy numbers of four copy unit alleles at AMY1 gene locus for trio samples are presented. The thin lines with labels NA12877 (father) and NA12878 (mother) represent copy numbers of them and the 11 dashed and dotted thin lines are that of their offspring. The thick line represents the sum of copy numbers of the parents. This result is consistent in a sense of heredity pattern in the trio samples, that is, copy number of offspring for each allele is less than or equal to the sum of that of its parents.

We identify copy unit sequences and copy numbers of each copy unit at AMY1 gene locus from 1123 samples. In this study we set the number of copy unit allele *K *to four which provides maximal lower bound of the log likelihood when varying *K *between one and 15. From estimated results, we confirmed that copy numbers of each allele for trio samples: NA12877 (father), NA12878 (mother), and their 11 offspring: NA12879, NA12880, NA12881, NA12882, NA12883, NA12884, NA12885, NA12886, NA12887, NA12888, and NA12893 are consistent in a sense of heredity pattern of diploid alleles indirectly (Figure 5), that is, estimated copy number of each allele for offspring is less than or equal to the sum of that of its parent samples (NA12877 and NA12878).

We also conducted the similar analysis for CHEK2P2, which is a pseudogene located at chrl5:20,487,996-20,496,839. The locus had 175 variable sites and its estimated copy numbers ranged from three to 12 in members of the CEPH/Utah pedigree 1463. The copy unit allele K was chosen as 12, which maximized the lower bound of the log likelihood when K was set from one to 15. The estimated copy numbers of haplotypes were consistent within family members, as similar to AMY1 locus.

## Conclusions

We proposed a novel computational approach to simultaneously infer copy unit alleles and their numbers in each sample at CNV loci from HTS data. We verified the prediction performance in estimating copy unit alleles in two different simulation analyses. In the simulation analysis 1, we prepared four alleles with 16 variable sites and succeeded to predict true number and sequences of prepared alleles by maximizing the lower bound of log likelihood. In the simulation analysis 2, we extracted known haplotype sequences from 45 males in CEU population and constructed artificial CNV alleles with lower-, middle-, and higher-copy numbers and varied depth of coverage. Although a dataset with higher copy numbers is more difficult for accurate estimation than with lower copy numbers, the approach achieved allele concordance > 0.9 in terms of precision, recall, and F-measure with HTS data of 10× mean depth of coverage per copy unit. We also applied the approach at highly variable salivary amylase gene locus and a pseudogene locus from HTS real data of 1123 samples that includes 17 high- and 1106 low-coverage alignment data. With this application, we confirmed consistency of inferred copy number for each allele of CEPH/Utah trio samples (NA12877, NA12878, and their 11 offspring).

We model copy numbers of copy unit alleles for each sample by relative amount of the alleles in the sample, instead of inferring combination of integer copy numbers of possible alleles explicitly which will be intractable for high copy number alleles due to the exponentially increasing number of possible states. Thanks to this feature, the computational complexity is linear order of number of alleles *K*, number of samples *N*, and number of variable sites *M *at CNV locus, as described in Methods section, and our approach is robust to increase in the number of alleles and samples.

Although this study presents a promising approach for CNV haplotyping from HTS data, there are several challenges beyond the current approach. First, utilizing full features of HTS data, such as base qualities, paired-end information, and cooccurrence of variable sites on single reads may improve the inference accuracy. Second, using or inferring the population history around CNV locus might improve the accuracy. However, it might be also needed to consider various events in the population history other than mutations such as duplications and recombinations around CNV loci and gene conversions [29,30], which will complicate the problem. Inference of diplotypes of CNV loci is also an important future work. Third, applying different approximation techniques such as a collapsed VB inference [25] or belief propagation [31] used for topic models of natural language processing to our model might improve accuracy of the inference.

## Competing interests

The authors declare that they have no competing interests.

## Authors' contributions

TM, NN, KK, and MN conceived the study. TM, NN, KK, and MN designed the computational experiments. TM performed the analysis, and TM, NN, KK, and MN interpreted the results. YS, YK, and YYK collaborated on data collection and interpretation of the results. TM, NN, KK, YS, YK, YYK and MN wrote the manuscript. All the authors read and approved the final manuscript.

## Declarations

The publication costs for this article were partly funded by MEXT Tohoku Medical Megabank Project.

This article has been published as part of *BMC Bioinformatics *Volume 16 Supplement 1, 2015: Selected articles from the Thirteenth Asia Pacific Bioinformatics Conference (APBC 2015): Bioinformatics. The full contents of the supplement are available online at http://www.biomedcentral.com/bmcbioinformatics/supplements/16/S1
